# Presence of bacterial DNA in synovial fluid from the temporomandibular joint in patients with temporomandibular joint disorders

**DOI:** 10.3389/froh.2026.1764272

**Published:** 2026-05-01

**Authors:** Nikoo Bazsefidpay, Mattias Ulmner, Anastasios Damdimopoulos, Bodil Lund

**Affiliations:** 1 Department of Oral & Maxillofacial Surgery, School of Medial Sciences, Faculty of Medicine and Health, Örebro University, Örebro, Sweden; 2 Head- Neck and Plastic Surgery Clinic, Department of Oral and Maxillofacial Surgery, Örebro University Hospital, Örebro, Sweden; 3 Division of Oral Diagnostics and Oral Surgery, Department of Dental Medicine, Karolinska Institute, Stockholm, Sweden; 4 Medical Unit of Plastic Surgery and Oral and Maxillofacial Surgery, Karolinska University Hospital, Stockholm, Sweden; 5 Bioinformatics and Expression Analysis Core Facility, Department of Medicine Huddinge, Karolinska Institute, Stockholm, Sweden

**Keywords:** bacteria, ribosomal, RNA, synovial fluid, temporomadibular joint, temporomandibular joint disorder

## Abstract

**Introduction:**

Temporomandibular joint disorders (TMJD) are common, affecting approximately 30% of the population. While the aetiology of TMJD has been considerably discussed, one aspect that has not been thoroughly investigated is the potential occurrence and role of bacteria in the temporomandibular joint (TMJ). This study aimed to determine the existence of bacteria in the TMJ synovial fluid of patients with TMJD.

**Methods:**

In this cohort study, synovial fluid from patients with TMJD, was collected during routine surgery at the Oral and Maxillofacial Unit at Karolinska University Hospital, Stockholm. Data analysis of the bacterial 16S rRNA sequencing was performed using automated cloud-based EPI2ME workflows (Oxford Nanopore Technologies).

**Results:**

The synovial fluid of 64 patients (mean age 41 years), of whom 86% (*n* = 55) were women, was analysed, and bacterial rRNA were detected in 64% (*n* = 41). Most commonly, one bacterial genus was detected in each patient (*n* = 19), two bacteria were detected in twelve patients, three in four patients, four in five patients and one patient had six different bacterial genera in its TMJ synovial fluid. The most common bacterial genera found were *Staphylococcus* (*n* = 8), *Streptococcus* (*n* = 7), *Cutibacterium* (*n* = 4), and *Peptoniphilus* and *Moraxella* (*n* = 3). The origin of these bacteria suggests two main dissemination routes to the TMJ: hematogenous spread and contamination during previous handling of sample.

**Conclusion:**

The findings of this study indicate that bacterial presence within the TMJ seems frequent and demonstrates diagnostic clustering, that warrant further investigation. However, the results should be interpreted with caution because of the high sensitivity of 16S rRNA sequencing.

## 1 Introduction

Temporomandibular disorders (TMD) refer to a spectrum of conditions affecting both the temporomandibular joint (TMJ) and masticatory muscles, impacting approximately 30% of the population (1, 2). Temporomandibular joint disorders (TMJD) can be divided into three main categories. The first is internal derangement, which involves the displacement of the TMJ disc. Disc displacement with reduction (DDwR) refers to a displaced disc when the jaw is in its resting position, most commonly in an anterior direction. When opening the jaw, the disc returns to its normal position, often accompanied by a clicking sound. If the disc is displaced in both closed and open mouth position, it is termed disc displacement without reduction (DDwoR). While DDwR patients are sometimes asymptomatic or have low grade of complaints and do not require treatment, patients with DDwoR often display symptoms such as pain and limited mouth opening necessitating intervention (3, 4, 6). The second group of TMJD includes inflammatory processes, such as various forms of chronic inflammatory arthritis (CIA), while the third encompasses degenerative disorders, such as osteoarthritis (OA), characterized by bone destruction (5, 6). Other TMJD include luxation, benign or malignant tumors, ankylosis and infection. A variety of surgical and nonsurgical treatments are available for these diagnoses (3, 6, 7).

The etiology of TMJD has been extensively discussed and is recognized as multifactorial, involving factors such as trauma, general joint hypermobility, bruxism, hormonal changes, and infection (8–12). One of the more controversial topics has been the role of bacteria in TMJD. Numerous studies have been conducted on other joints and prosthesis infections, as well as the presence of bacteria in the synovial fluid of patients with arthritis (13–17). It has been suggested that bacterial infection of the TMJ can occur due to hematogenous spread of bacteria, trauma, surgery, or infection spread from nearby structures such as otitis externa (18, 19). Most studies on this subject have focused on TMJ septic arthritis (20, 21). However, few studies have explored the role of bacteria in patients with different TMJD, and the results have been varied. A study by McIntosh and Dimitroulis investigated the presence of bacteria in 12 fresh TMJ retrodiscal tissue samples. The tissue samples were stained and cultured, and no bacteria was found (22). Henry et al. found *C trachomatis in* 29% of bilaminar tissues samples in his study using PCR analysis (23). Some studies have also employed bacterial 16S rRNA sequencing in TMJ synovial fluid, including one by Kim et al., who detected bacteria in both healthy and TMJD groups (24). In contrast, Wei et al. used the same methodology and found bacteria only in the synovial fluid of the TMJD group (19).

There are many different methodologies for detecting bacteria, each offering different levels of sensitivity. The most common method used in hospitals is conventional culture, which requires a sufficient bacterial load and may overlook some bacteria (25). During recent decades, 16S rRNA gene sequencing has gained popularity in both clinical and research settings due to its ability to detect a wide range of bacteria, including not-yet-culturable bacteria. However, it is highly susceptible to contamination and can identify environmental and background bacteria (26–28). Another approach is species-specific PCR, known for its high sensitivity and specificity for target bacteria (29, 30). Yet, in the context of TMJD, where knowledge of the existing bacteria is limited, identifying targeted bacteria can be challenging, potentially leading to the oversight of other significant ones.

This study aimed to investigate the presence of bacterial DNA in the TMJ synovial fluid of patients with internal derangements, chronic inflammatory arthritis, or osteoarthritis using 16S rRNA gene sequencing.

## 2 Material and methods

### 2.1 Study design and patient selection

This cohort study included patients treated at Karolinska University Hospital, Stockholm, Sweden, between December 2014 and January 2017. The inclusion criteria were patients with TMJD: DDwR, DDwoR, OA, and CIA, where the latter includes diagnoses such as rheumatoid arthritis, psoriatic arthritis, and spondylarthritis. The patients had undergone at least three months of non-invasive therapy and had a visual analog scale (VAS) score of ≥4 for TMJ pain and function. Patients with DDwoR had to show a maximum interincisal opening (MIO) ≤ 35 mm to be included. Synovial fluid was sampled during the operation. The exclusion criteria were age <18 years, history of open joint surgery, and inability to provide informed consent.

The cohort was initially collected prospectively where the clinical data, synovial fluid, and tissue samples were analyzed for cytokine presence (ethical approval 2014/622-31/1) (31). An additional ethical approval (2021-03618) was obtained to conduct 16S rRNA analysis on the remaining synovial fluid.

### 2.2 Synovial fluid sampling

The synovial fluid was collected by a single surgeon during TMJ arthroscopy or discectomy. Using the push and pull method described by Alstergren et al., synovial fluid was collected prior to any penetration of the TMJ capsule under sterile conditions (32, 33). A solution containing saline and vitamin B12 was used to wash the intraarticular joint space four times, and then the synovial fluid was collected. The samples were directly stored at −80°C, after determination of the ratio of synovial fluid in the samples. No prophylactic antimicrobial treatment was administered preoperatively to any of the patients.

### 2.3 Analysis of bacterial DNA in synovial fluid

After an enzymatic pretreatment with Mutanolysin (100U, 1-hour incubation at 37 °C), microbial DNA was extracted from 200 µL of the samples using the MagDea Dx Sv kit [Precision System Science (PSS), Matsudo, Chiba, Japan] on a MagLEAD 12gC automated system (PSS). The purified DNA was eluted in 50 µL nuclease-free H_2_O.

The full-length 16S rRNA gene (approximately 1,500 bp, covering variable regions V1–V9) was sequenced to identify bacterial organisms at the species level. PCR amplification and preparation of barcoded DNA libraries for sequencing were performed with the 16S Barcoding Kit [SQK-16S024, Oxford Nanopore Technologies (ONT), Oxford, United Kingdom], following the manufacturer's instructions, but with an increased number of PCR cycles from 25 to 40. The number of PCR cycles was increased to enhance sensitivity in low-biomass samples. However, this may also increase the risk of amplifying background contamination, which was addressed through negative controls and strict exclusion criteria. DNA libraries were quantified using the Qubit 1X dsDNA HS Assay Kit on a Qubit 3.0 fluorometer (Thermo Fisher, Waltham, MA, USA) and were pooled in equal volumes rather than equimolar concentrations prior to sequencing on a R9.4.1 flowcell on a GridION platform (ONT).

Sequencing was performed for 24 h, and unlike short-read platforms with predefined target depth, Nanopore sequencing generates data continuously over the runtime. As libraries were pooled in equal volumes and not normalized to equimolar concentrations, sequencing depth varied between samples, and the total read count per sample reflects both biological DNA load and technical variation in library preparation, amplification and sequencing.

Basecalling was performed using Guppy (v5.1.13), followed by demultiplexing with removal of barcode and adapter sequences. Quality filtering was carried out using NanoFilt (v2.8), keeping reads with a quality score >9 and lengths between 1,400–18,00 bp. Taxonomic assignment was performed using the Oxford Nanopore EPI2ME 16S workflow as well as Kraken2, a widely used k-mer—based classifier for microbial identification. The results, including quality control metrics and taxonomic profiles, were compiled into summary reports. These outputs provided a comprehensive overview of the bacterial composition in each sample. The relative abundance of reads for each sample were evaluated. Hits above a three percent relative abundance was further evaluated and reported at the genus level.

### 2.4 Contamination control

Synovial fluid samples were collected and handled in a sterile environment in the operating room and laboratory to minimize the risk of contamination. A negative control containing saline and vitamin B12, in the exact same amounts used initially to collect the synovial fluid, was added to each batch to control DNA contamination and cross-contamination. A commercially available mock microbial community (ZymoBIOMICS™ Gut Microbiome Standard, D6331, Zymo Research, USA), consisting of defined bacterial species with known composition, was used as a positive control to monitor DNA extraction efficiency, amplification, and sequencing performance.

### 2.5 Statistical analysis

All statistical analyses were performed in R (version 4.5; R Foundation for Statistical Computing, Vienna, Austria). Abundance and count matrices were imported and pre-processed within the R environment. Data visualization was conducted utilizing ggplot2 for general plotting, VennDiagram for Venn diagram generation, and ComplexHeatmap for heatmap representation. Diversity metrics and ecological indices were calculated using the vegan package. All packages were obtained via CRAN or Bioconductor repositories.

## 3 Result

### 3.1 Patients' characteristics

Synovial fluid was analyzed from 64 of the 93 patients included in the original study, the remaining 29 had no synovial fluid available for further analysis. The demographic data is presented in Table 1. The TMJ diagnoses were as follows: DDwoR 48% (*n* = 31), DDwR 16% (*n* = 10), OA 17% (*n* = 11), and CIA 19% (*n* = 12). The mean age of included patients were 41 years old with a female predominance (86%). Twenty-two percent (*n* = 14) of the patients were otherwise healthy, while the largest group of medical conditions were psychiatric 30% (*n* = 19), or autoimmune diseases 23% (*n* = 15).

**Table 1:** Demographic data of the included patients, and bacterial genus in TMJ synovial fluid.

Characteristics	All patients	DDwoR	DDwR	OA	CIA
Number of patients, *n* (%)	64 (100)	31 (48)	10 (16)	11 (17)	12 (19)
Patient gender, *n* (%)					
Male	9 (14)	6 (67)	3 (33)	0 (0)	0 (0)
Female	55 (86)	25 (45)	7 (13)	11 (20)	12 (22)
Patient age, years, *n* (%)					
0–19	3 (5)	1	0	1	1
20–39	29 (45)	14	6	4	5
40–59	24 (38)	11	4	3	6
>60	8 (12)	5	0	3	0
Mean age (SD)	41 (15.8)	41 (16.2)	37 (10.9)	46 (22.0)	38 (12.3)
Medical condition^a^					
Healthy	14 (22)	8	5	1	0
Psychiatric	19 (30)	12	3	2	2
Neuropsychiatric	3 (5)	1	1	0	1
Autoimmune	15 (23)	1	0	2	12
Metabolic	5 (8)	3	1	1	0
Cardiovascular	8 (13)	3	1	3	1
Cancer	1 (2)	1	0	0	0
Other^b^	33 (52)	17	3	7	7
Bacteria in TMJ synovial fluid, *n* (%)					
Presence	41 (64)	17 (41)	6 (15)	9 (22)	9 (22)
Absence	23 (36)	14 (61)	4 (17)	2 (9)	3 (13)

CIA, chronic inflammatory arthritis; DDwoR, disc displacement without reduction; DDwR, disc displacement with reduction; OA, osteoarthritis.

a Note, an individual patient can have several diagnoses, explaining the distributed number exceeding total patient population size.

b Other: asthma, general osteoarthrithis, migrane, cluster headchae, adenomyoma, endometriosis etc.

### 3.2 Bacterial presence

The results from the initial bioinformatic analysis identified reads matching 820 bacterial genera from the reference database. Species-level classification was generated, but results were aggregated at the genus level to reduce uncertainty associated with species-level assignments. Each batch result was compared to the negative control of the batch. Several bacterial genera were detected in the negative controls, with varying read counts. Any genus detected in a negative control was excluded from the reported results, regardless of read count. This conservative filtering strategy excluding all taxa present in negative controls, was applied to further minimize overinterpretation. Thus, batch-to-batch variation likely reflects a combination of reagent-derived contaminants, environmental DNA, and stochastic amplification effects inherent to low-biomass samples, particularly with increased PCR cycle numbers. The top 20 bacterial genera detected in the negative controls for each batch, including read counts, are presented in Supplementary Figure 1. A cut-off at 3% relative abundance was established with the assistance of a bioinformatic expert, supported by batch-specific elbow-curve assessments of read-count distributions (Supplementary Figure 2) and individual sample cutoff calculations showing a median of 3.2% (Supplementary Figure 3). These analyses indicated a distinct inflection at this level across sequencing batches. These cut-offs were applied, resulting in a total of 50 bacterial genera identified.

**Figure 1:**
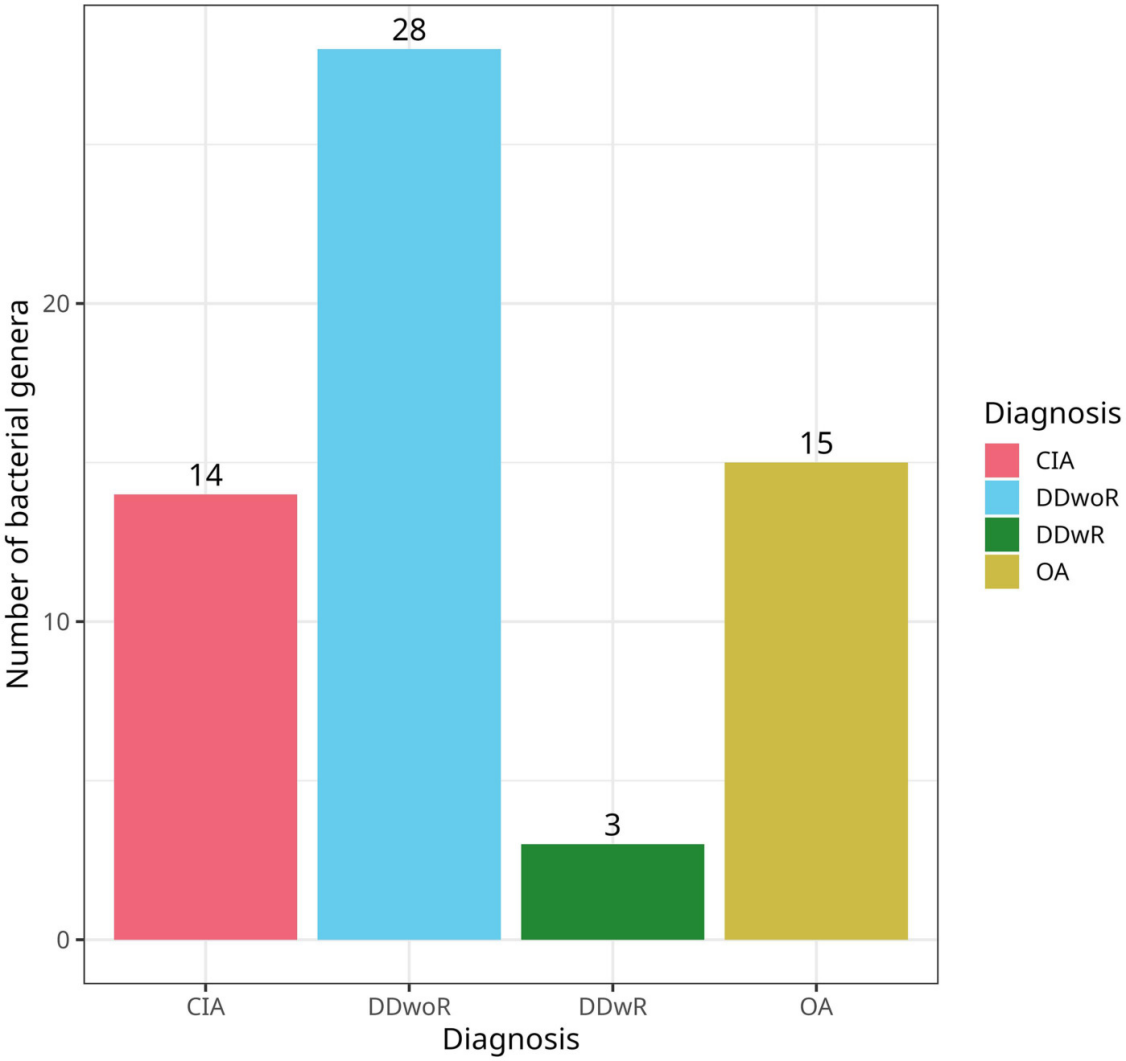
Bar charts showing bacterial genus counts related to diagnosis. Number of bacterial genera in each diagnostic group. Bacterial genera with less than 3% relative abundance and if present in the negative controls of the same batch were removed during contamination filtering. CIA, chronic inflammatory arthritis; DDwoR, disc displacement without reduction; DDwR, disc displacement with reduction; OA, osteoarthritis.

**Figure 2:**
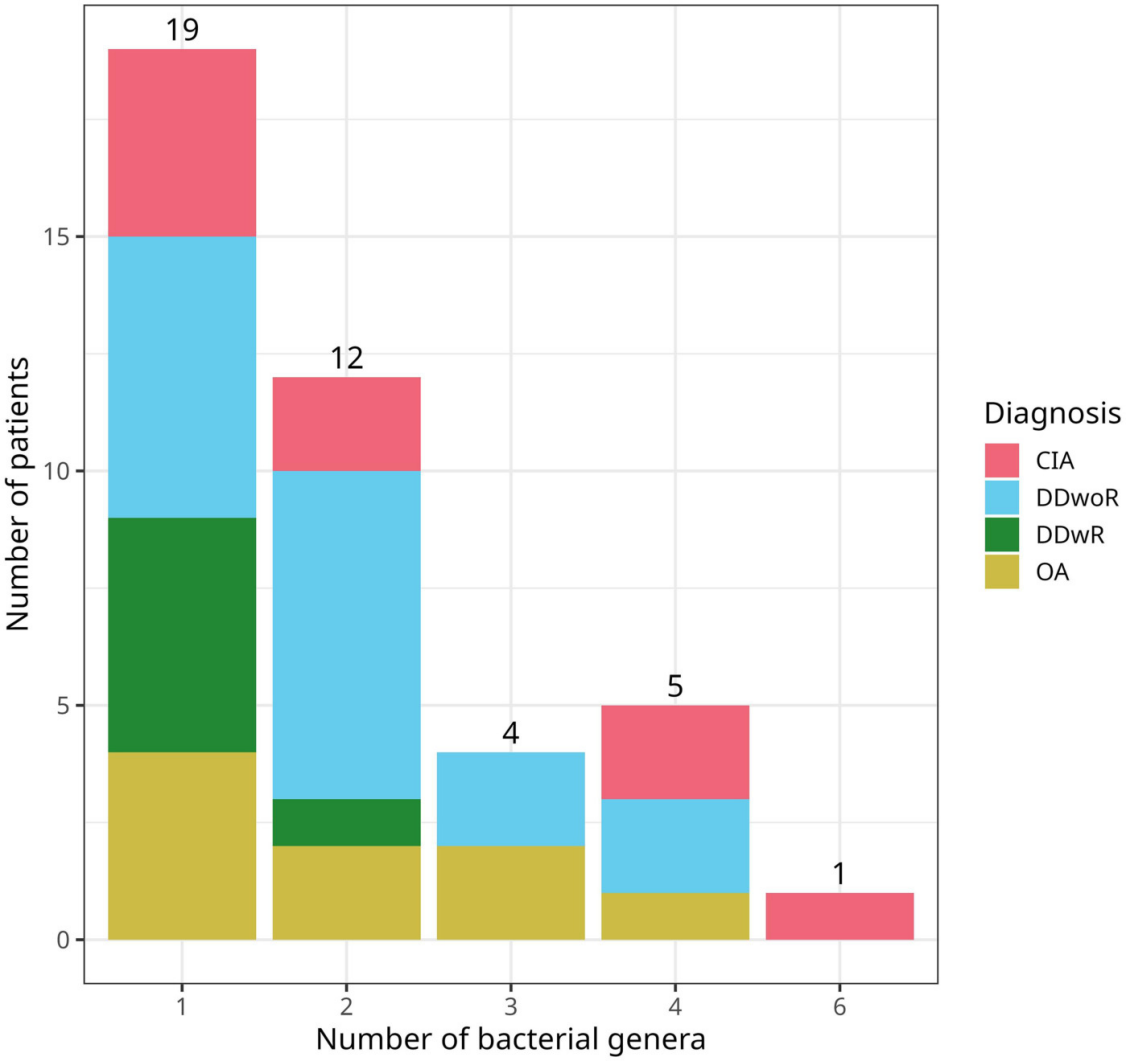
Number of patients grouped by the number of bacterial genera detected in each patient and coloured by diagnosis. Bacterial genera with less than 3% relative abundance and if present in the negative controls of the same batch were removed during contamination filtering. CIA, chronic inflammatory arthritis; DDwoR, disc displacement without reduction; DDwR, disc displacement with reduction; OA, osteoarthritis.

Among the 64 patients, 64% (*n* = 41) had bacteria present in the TMJ synovial fluid, while 36% (*n* = 23) showed no bacterial genus (Table 1). Most patients had a single bacterial genus (*n* = 19), with one patient exhibiting six different bacterial genera in the TMJ synovial fluid. Figure 1 displays the bacterial presence by the number of bacteria relative to the patient number, categorized by each diagnosis. Looking closer into the diagnoses, the highest ratios (bacterial genera/ total number) occurred in OA (*N* = 15, ratio 1.36) and CIA (*n* = 14, ratio 1.17). Meanwhile DDwoR had highest number of bacteria *n* = 28 with ratio 0.90 and DDwR (*n* = 3, ratio 0.30) had the lowest (Figure 2).

**Figure 3:**
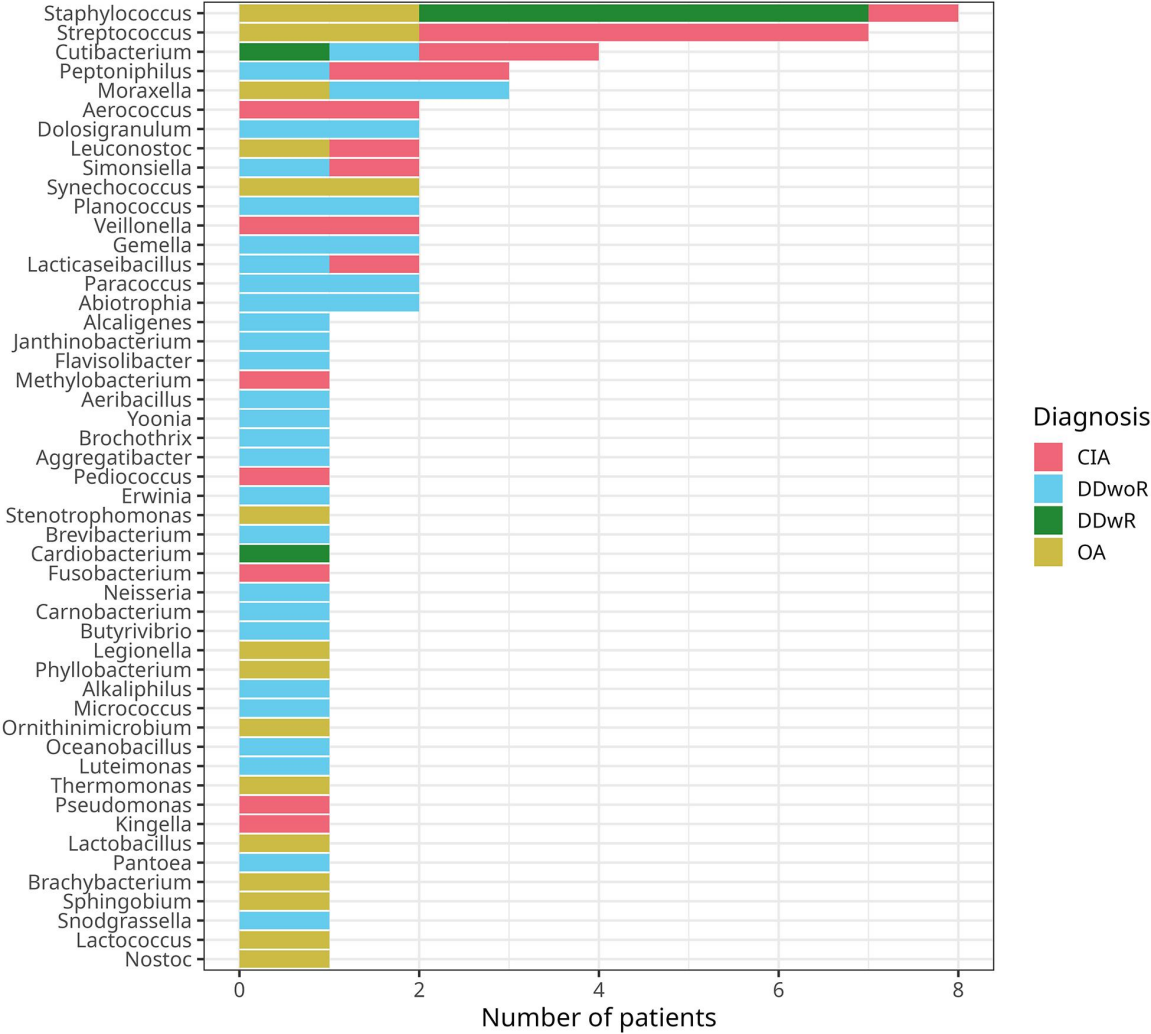
Bacterial Genera detected and their prevalence among patients with different diagnosis. Number of patients in which each bacterial genus was detected in, split by diagnosis which is indicated by colour.Bacterial genera with less than 3% relative abundance and if present in the negative controls of the same batch were removed during contamination filtering. CIA, chronic inflammatory arthritis; DDwoR, disc displacement without reduction; DDwR, disc displacement with reduction; OA, osteoarthritis.

**Figure 4:**
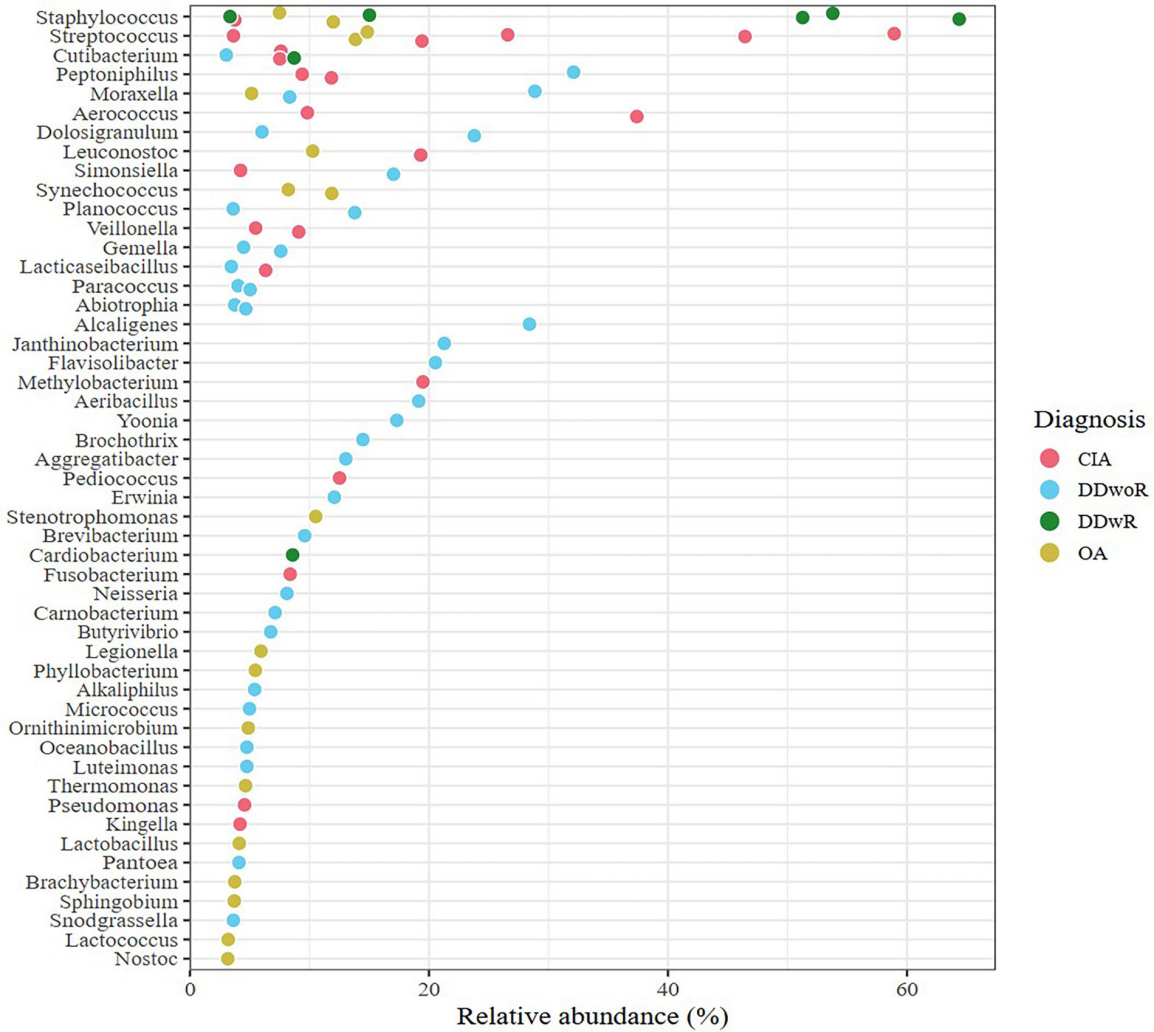
The relative abundance of each bacterial genus in individual patients. Each point is one patient, where the diagnosis is colour coded. Bacterial genera with less than 3% relative abundance and if present in the negative controls of the same batch were removed during contamination filtering. CIA, chronic inflammatory arthritis; DDwoR, disc displacement without reduction; DDwR, disc displacement with reduction; OA, osteoarthritis.

Most of the 50 bacterial genera identified in TMJ synovial fluid, were found in one patient only (68%, *n* = 34). The most common bacterial genus was *Staphylococcus*, found in eight patients, followed by *Streptococcus* in seven, *Cutibacterium* in four, and *Peptoniphilus* and *Moraxella* in three patients each (Figure 3). The number of bacterial genera present in each sample (relative abundance) divided by diagnosis is shown in Figure 4. The origin of all the bacterial genera present in ≥2 patients is detailed in Table 2.

**Table 2:** Bacterial genus characteristics and where they are predominantly found, detected in two or more patient samples.

Name of the bacteria genus	Characteristics	Predominantly found
*Staphylococcus*	Gram-positive cocci; facultative anaerobes	Skin and mucous membranes
*Streptococcus*	Gram-positive cocci; facultative anaerobes	Mouth, throat, skin, and respiratory tract.
*Cutibacterium*	Gram-positive anaerobic rods	Skin, sebaceous glands
*Peptoniphilus*	Gram-positive anaerobic cocci	Respiratory tract
*Moraxella*	Gram-negative diplococci; aerobic	Respiratory tract
*Aerococcus*	Gram-positive cocci; facultative anaerobes	Air, dust, and occasionally human skin and urinary tract
*Dolosigranulum*	Gram-positive cocci; facultative anaerobes	Nasopharynx
*Leuconostoc*	Gram-positive cocci; facultative anaerobes	Fermented foods, plants, and occasionally human flora
*Simonsiella*	Gram-negative rods; aerobic	Oral cavity of mammals, rarely studied in humans; part of normal oral flora
*Synechococcus*	Gram-negative cyanobacteria; photosynthetic	Marine and freshwater
*Planococcus*	Gram-positive cocci; aerobic	Marine, soil, and saline environments
*Veillonella*	Gram-negative anaerobic cocci	Oral cavity, gastrointestinal and respiratory tracts.
*Gemella*	Gram-positive cocci; facultative anaerobes	Oral cavity, upper respiratory, and gastrointestinal tracts
*Lacticaseibacillus*	Gram-positive rods; facultative anaerobes	Fermented foods and human gastrointestinal tract.
*Paracoccus*	Gram-negative coccoid or rod-shaped bacteria; aerobic	Soil and water
*Abiotrophia*	Gram-positive cocci; facultative anaerobes	Oral cavity, respiratory tract, genitourinary tract; nutritionally fastidious

**Figure 5:**
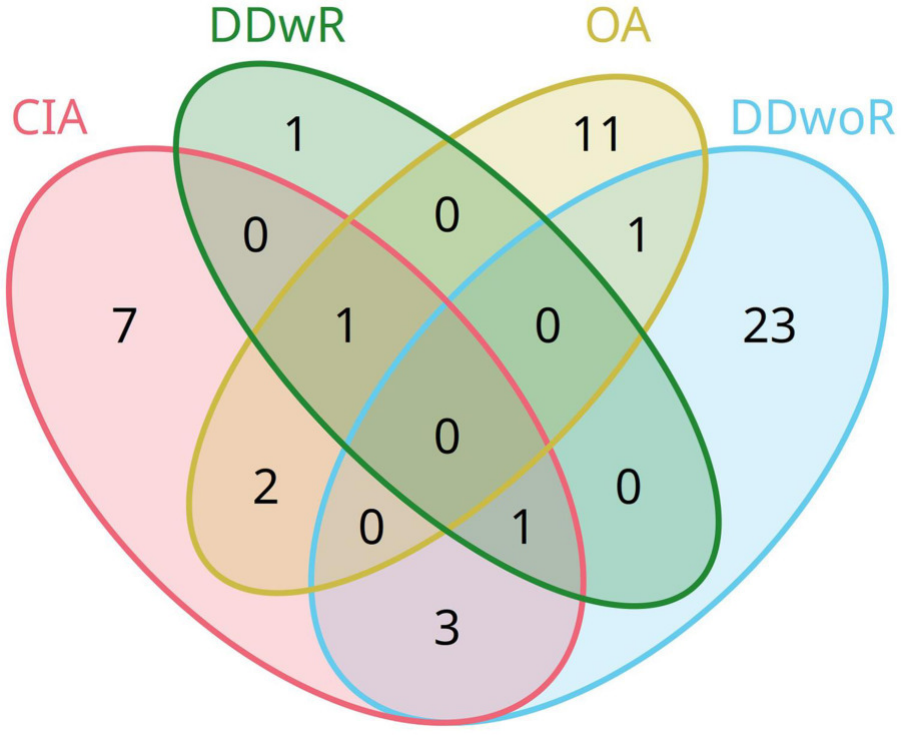
Venn diagram showing overlapping and unique bacterial genera detected across diagnostic groups. Bacterial genera with less than 3% relative abundance and if present in the negative controls of the same batch were removed during contamination filtering. CIA, chronic inflammatory arthritis; DDwoR, disc displacement without reduction; DDwR, disc displacement with reduction; OA, osteoarthritis.

The presence of individual bacterial genera across diagnostic groups was assessed to determine patterns of overlap. Figure 5 depicts the extent of genus-level overlap among the four diagnoses. No bacterial genus was identified as common across all diagnostic categories. However, *Cutibacterium* was found in CIA, DDwoR, and DDwR patients and *Staphylococcus* in CIA, DDwR, and OA group. Three genera were shared exclusively between patients diagnosed with CIA and DDwoR (*Simonsiella, Peptoniphilus,* and *Lacticaseibacillus*), two genera overlapped between CIA and OA (*Streptococcus* and *Leuconostoc*), and one genus was present in patients with concurrent diagnoses of OA and DDwoR (*Moraxella*) (Figure 5).

**Figure 6:**
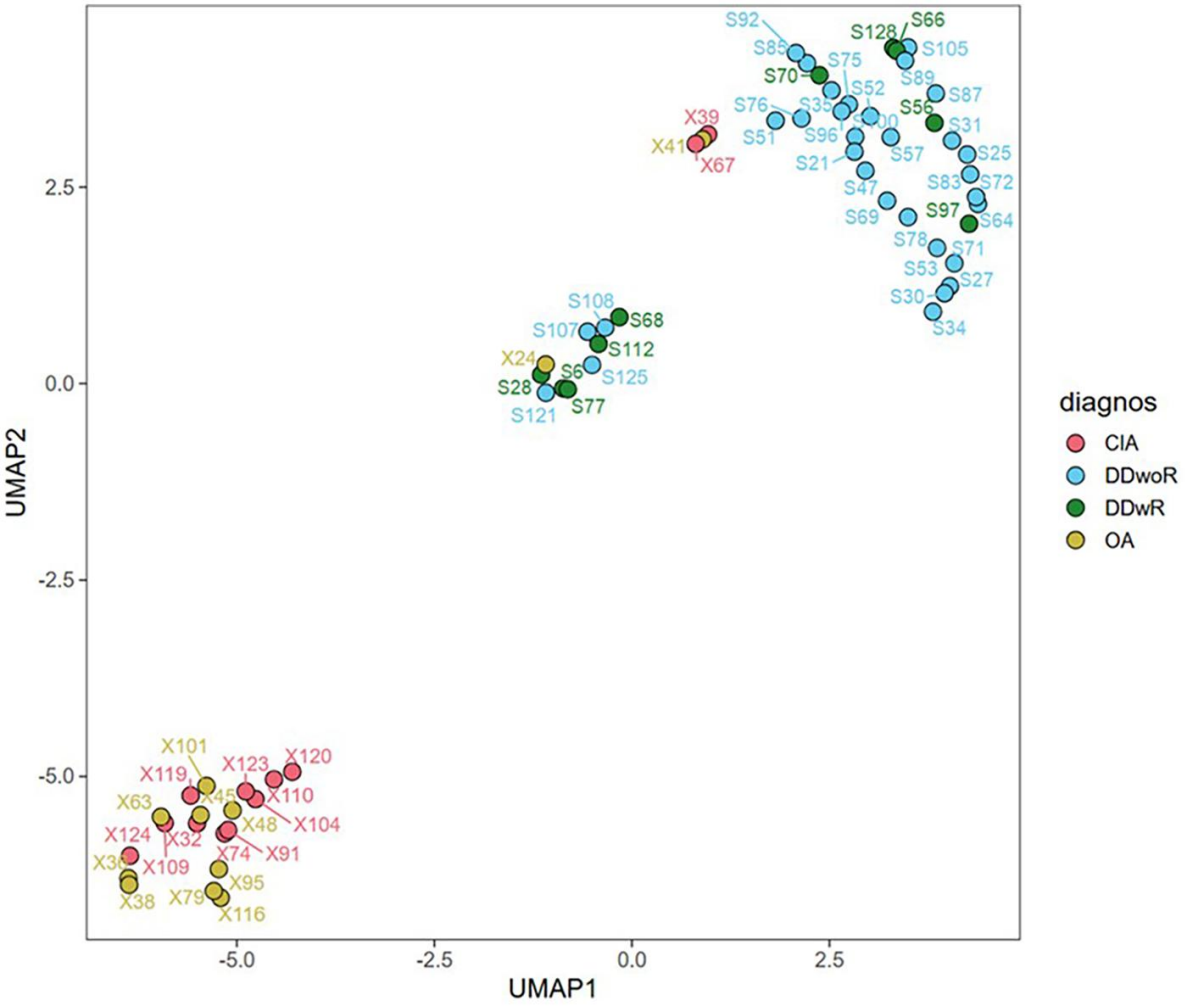
Each point is a patient, divided into different diagnosis indicated by a specific colour. The UMAP was done on bionomial data. 1 if bacteria were present, 0 if not. Distinct clusters for each diagnosis are visible, with groups separated in different quadrants. DDwoR, disc displacement without reduction; DDwR, disc displacement with reduction; OA, osteoarthritis; CIA, chronic inflammatory arthritis.

Finally, to explore potential similarity, pattern, and cluster within the diagnosis, Uniform Manifold Approximation and Projection (UMAP) was used. The binomial data, with one (1) indicating the presence of a bacterial genus and zero (0) indicating its absence, suggested a tendency for cluster of diagnoses DDwoR with DDwR and OA with CIA (Figure 6). Similar results are shown if the UMAP is plotted on normalized count and counts per million.

## 4 Discussion

The aim of this study was to investigate the presence of bacterial DNA in TMJ synovial fluid, which was detected in a relatively large proportion of the patients studied. Since joints are normally considered as sterile environments, these findings are noteworthy. Presence of bacteria of low virulence not giving rise to infection may have the potential to cause, and maintain, inflammatory processes and symptoms. If these microorganisms are the main drivers of the conditions, or merely bystanders with the potential of potentiating an already existing condition remains to be proven.

The origin of the bacteria is of relevance because it gives clues of the route of transmission to the joint but also because it might open for potential preventive measures. Therefore, considering the detected species in relation to their normal habitat may provide useful information. It can be hypothesized that there are two main bacterial dissemination tracts to the TMJ, one being hematogenous spread via the bloodstream, facilitated by the highly vascular synovium, and the other through penetration of the skin, iatrogenic or by trauma. Consequently, occurrence of certain bacteria originating from the skin, such as *Staphylococcus* and *Cutibacterium*, might be explained by recruitment of bacteria from the blood stream, but contamination during the surgery cannot be excluded. Meanwhile, bacteria like *Streptococcus*, *Simonsiella*, *Veillonella*, and *Gemella* originate from the oral cavity, while *Peptoniphilus* and *Lacticaseibacillus* come from the gastrointestinal tract and may spread hematogenously in the highly vascular synovium of the TMJ (34). Previous studies have identified some of these bacteria. Kim et al. found *Mycoplasma genitalium* (86%), *Staphylococcus aureus* (51%), *Mycoplasma fermentans/orale* (37%), *Actinobacillus actinomycetemcomitans* (26%), and *Streptococcus* (7%) in TMJ synovial fluid samples (24). In an investigation of the posterior bilaminar tissue, *Chlamydia trachomatis* was detected in 20% of patients (23). In another study using the same methodology (PCR 16s rRNA), 11 bacterial species were identified in 68% of their patients (19). Finally, Olsen-Bergem et al. also found some of the same bacteria as this study in more than 50% of patients’ TMJ synovial fluid (18). These findings suggest that low-grade inflammation driven by microorganisms could be a contributing factor in the pathogenesis of TMJD. Patient specific factors including periodontal disease, gastrointestinal dysbiosis, and systemic inflammatory conditions prevalent among TMJD cohorts may facilitate hematogenous seeding of synovial fluid. In addition, it cannot be excluded that bacterial DNA may be transported to the joint by macrophages or other leukocytes, which could contribute to the presence of bacterial DNA without viable bacteria in the synovial fluid. However, these interpretations remain speculative without control group data or longitudinal sampling to distinguish transient contamination from persistent colonization. Future studies employing paired blood cultures, surgical skin swabs, and salivary sampling could help differentiate these pathways and clarify their clinical significance. Furthermore, the diversity UMAP illustrates the clustering patterns in patients diagnosed with DDwoR and DDwR, and CIA and OA. This suggests similarities within these two clusters regarding diagnoses and the bacterial profile and occurrence, potentially reflecting common underlying pathophysiological mechanisms or environmental factors (Figure 6). Although, the clustering might be by chance since the number of patients in each diagnostic group was small, implicating that investigations with larger patient populations is advocated.

This study identified 50 different bacterial genera, and it is important to interpret the result with caution. Some of these bacteria are also typically found in laboratories, air, dust, and food, and are occasionally detected as contaminants. Currently, PCR and 16S rRNA sequencing are routinely employed in both clinical and research settings and strict sterile routines are well established. However, these highly sensitive techniques can detect microbiomes even at very low concentrations, which can easily lead to overinterpretation of the results (28, 35–37). A careful and data based cut off for detection limits is crucial and has been applied in the current study. A limitation of this study is that the operations were conducted in a single hospital, where the samples were also initially processed. On a later occasion, microbiological analyses were carried out in a different hospital laboratory. Despite sterile conditions, factors such as transport, the involvement of multiple personnel, and the laboratory environment possibly might have influenced the findings. However, the fact that a part of the samples remained negative in terms of bacterial findings (36%) supports the assumption that the contamination preventive measures were sufficient.

Further analysis of bacterial taxa detected in the negative controls revealed that different taxa appeared across sequencing batches and that their read counts varied (Supplementary Figure 1). Although taxa with very low read counts in negative controls but higher abundance in patient samples were initially considered for retention, a conservative approach was ultimately adopted, excluding any taxa detected in negative controls regardless of read count, in order to minimize the risk of overinterpretation.

The results from the present study showed that presence of bacteria in the TMJ was not uncommon and displays clustering in regards of diagnoses, which could indicate a possible association with these conditions. In nine of twelve patients with a diagnosis of CIA and in nine of eleven patients with a diagnosis of AO, bacterial DNA was present, and these diagnoses are often associated with inflammation. Meanwhile, six out of ten patients with the disc-related diagnosis DDwR, and 17 out of 31 with DDwoR had bacterial DNA in the synovial fluid. There have been very few studies in this field, and larger prospective studies, ideally including a control group of patients without TMJD disorders, are needed to reach definitive conclusions. Given the descriptive nature of this study with a modest sample size and diagnostic group imbalances, firm diagnostic, prognostic, or therapeutic conclusions remain premature. However, the microbial patterns observed merit further investigation into their potential clinical relevance. The present findings can serve as a foundation for further research, enhancing our understanding of the etiology behind different TMJD, potentially influencing future prevalence estimates and subsequently open for more individualized treatment options.

## Data Availability

The next-generation sequencing data supporting the findings of this study have been deposited as unassembled FASTQ files in the NCBI Sequence Read Archive under BioProject ID: PRJNA1347794 with BioSample accessions: SAMN52869509-SAMN52869582.
