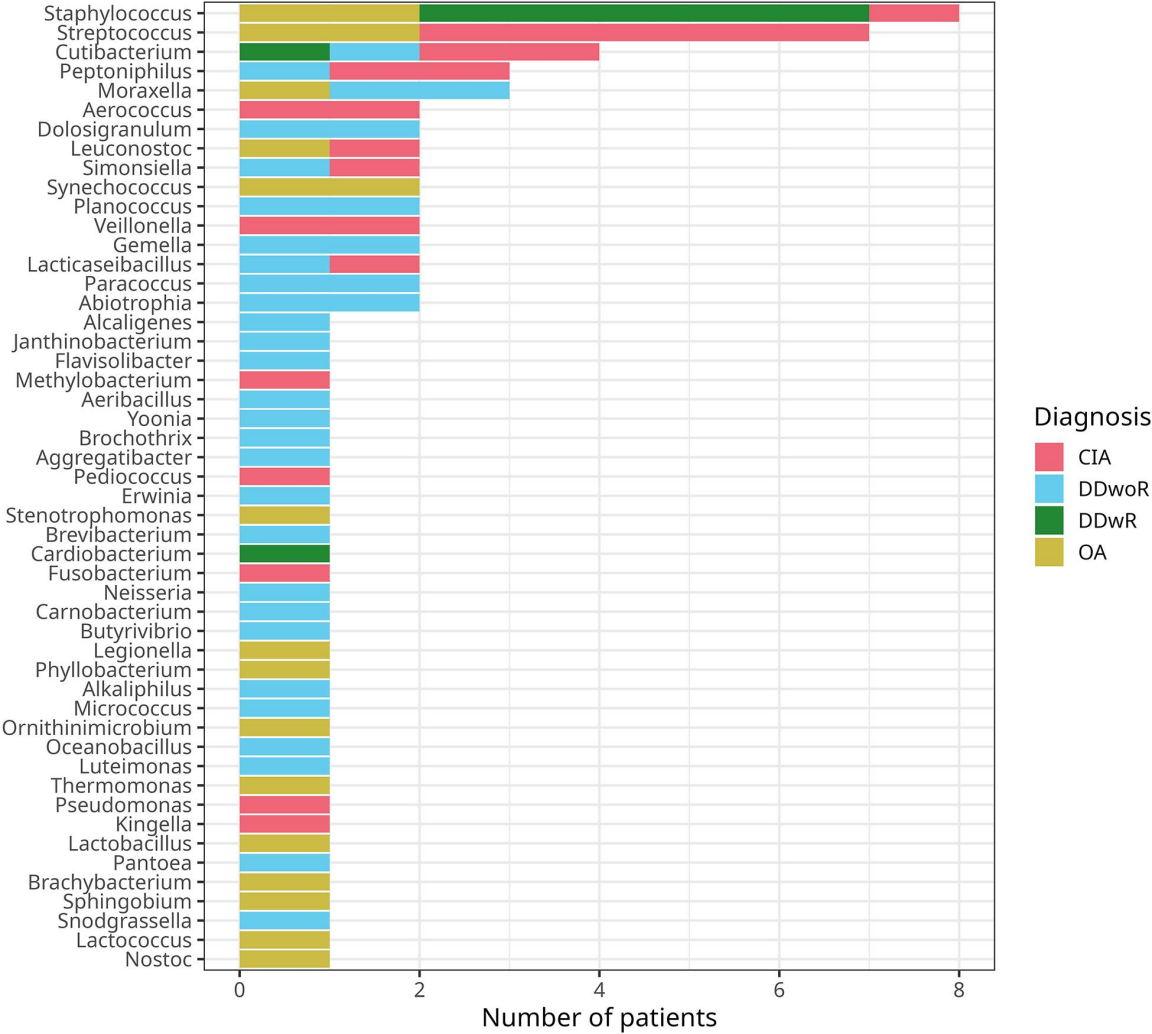# Correction: Presence of bacterial DNA in synovial fluid from the temporomandibular joint in patients with temporomandibular joint disorders

**DOI:** 10.3389/froh.2026.1881532

**Published:** 2026-06-22

**Authors:** Nikoo Bazsefidpay, Mattias Ulmner, Anastasios Damdimopoulos, Bodil Lund

**Affiliations:** 1 Department of Oral & Maxillofacial Surgery, School of Medial Sciences, Faculty of Medicine and Health, Örebro University, Örebro, Sweden; 2 Head- Neck and Plastic Surgery Clinic, Department of Oral and Maxillofacial Surgery, Örebro University Hospital, Örebro, Sweden; 3 Division of Oral Diagnostics and Oral Surgery, Department of Dental Medicine, Karolinska Institute, Stockholm, Sweden; 4 Medical Unit of Plastic Surgery and Oral and Maxillofacial Surgery, Karolinska University Hospital, Stockholm, Sweden; 5 Bioinformatics and Expression Analysis Core Facility, Department of Medicine Huddinge, Karolinska Institute, Stockholm, Sweden

**Keywords:** bacteria, ribosomal, RNA, synovial fluid, temporomadibular joint, temporomandibular joint disorder

There was a mistake in Figure 3 as published. The wrong version of Figure 3 was published. The corrected Figure 3 appears below.

The original version of this article has been updated.

**Figure 3:**