# Supplementary figures and images for: Presence of bacterial DNA in synovial fluid from the temporomandibular joint in patients with temporomandibular joint disorders
Source: Front Oral Health. 2026 May 1;7:1764272. doi: 10.3389/froh.2026.1764272 (PMC13176315; doi:10.3389/froh.2026.1764272)

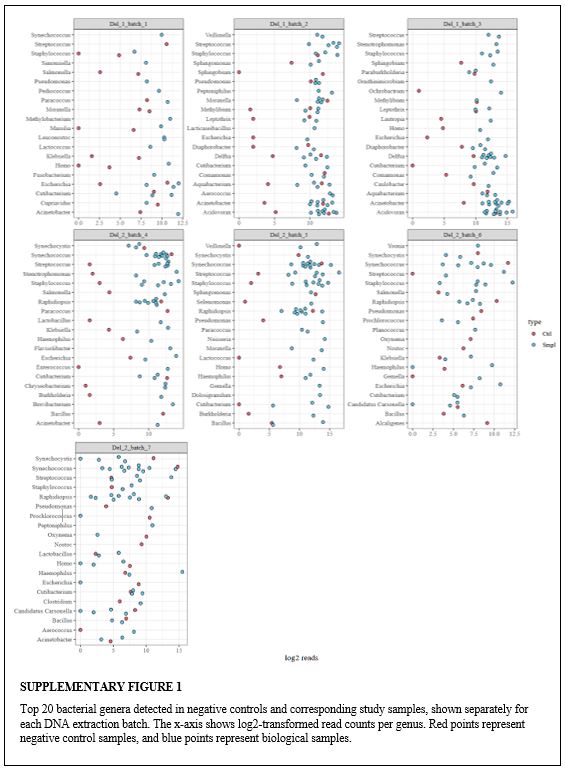

Supplement: Supplementary file 1 [file Image1.jpeg]

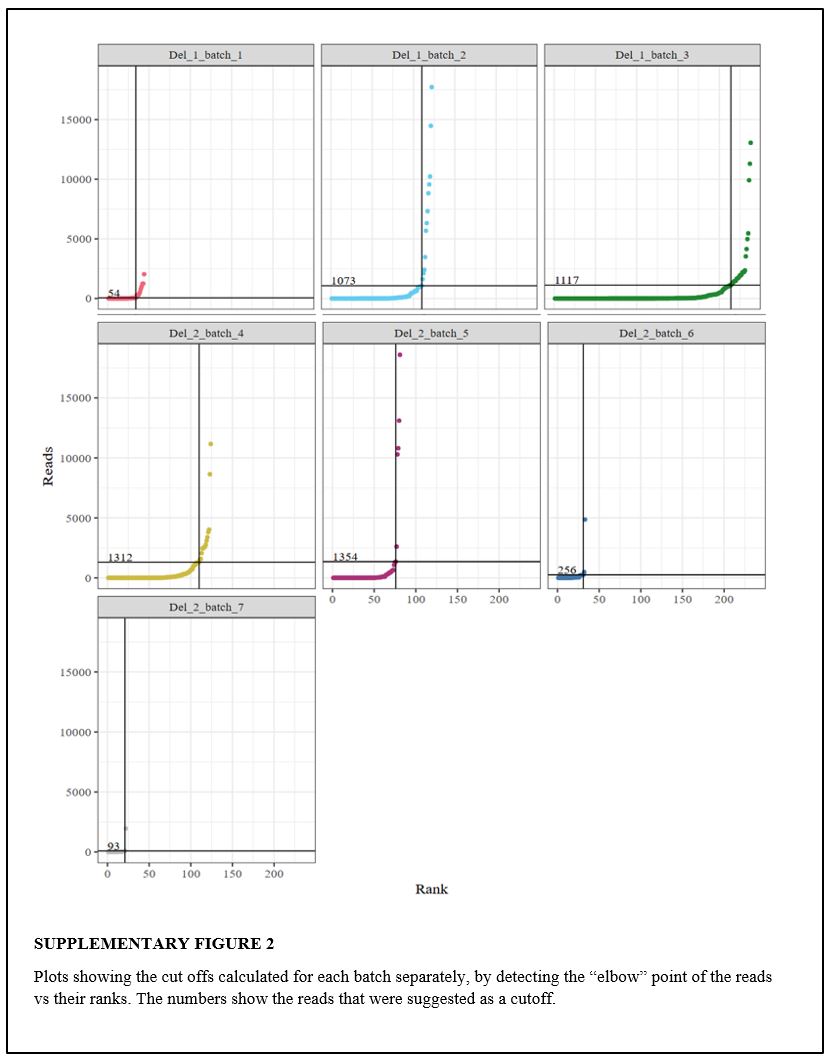

Supplement: Supplementary file 2 [file Image2.jpeg]

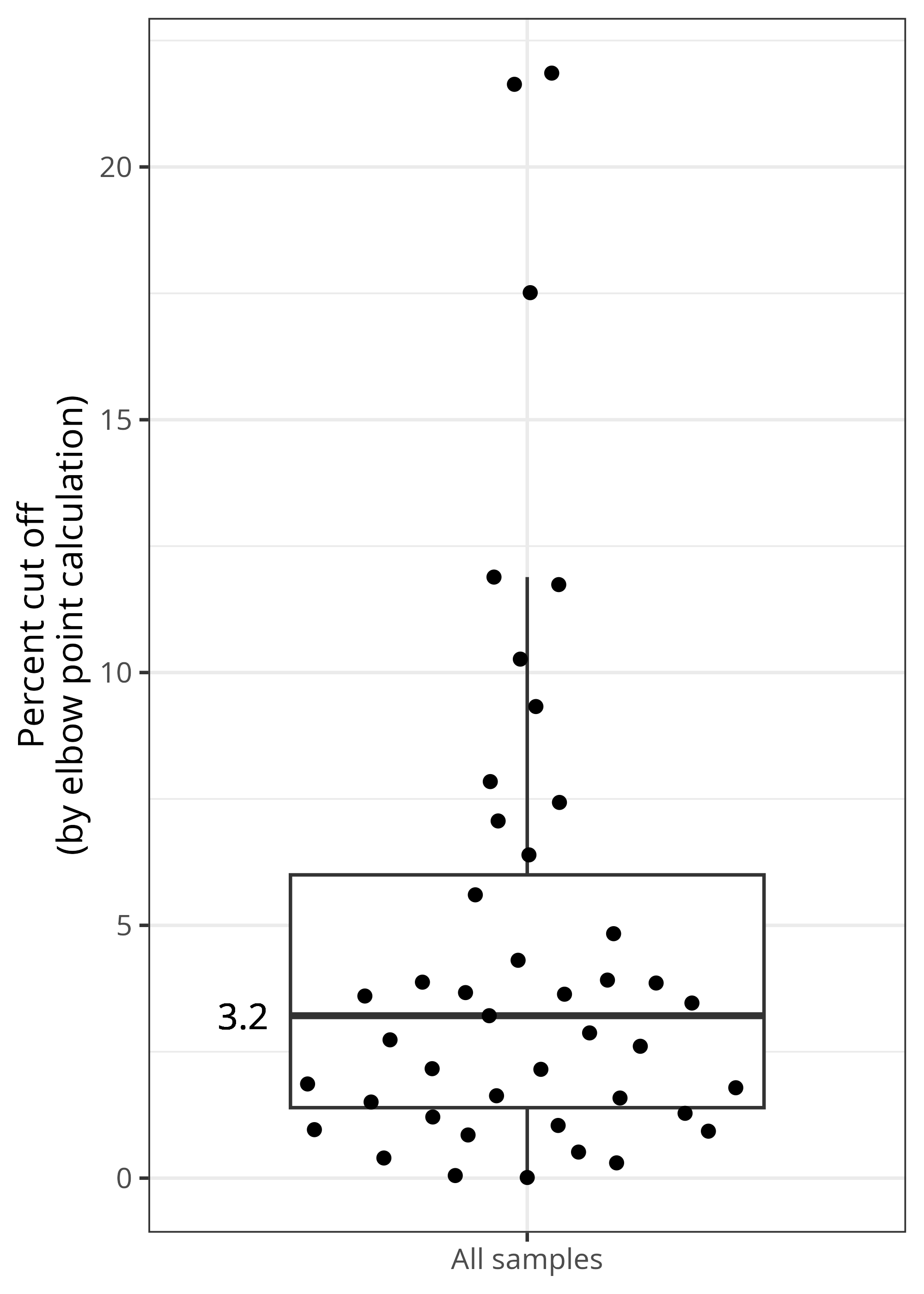

Supplement: Supplementary file 3 [file Image3.png]
